# Supplementary material for: A population genetic analysis of the nematode Strongyloides stercoralis in Asia shows that human infection is not a zoonosis from dogs
Source: Proc Natl Acad Sci U S A. 2025 Jul 15;122(29):e2424630122. doi: 10.1073/pnas.2424630122 (PMC12304889; doi:10.1073/pnas.2424630122)
Supplement: Supplementary file 1 — Appendix 01 (PDF) [file pnas.2424630122.sapp.pdf]

Supplementary Methods

Parasite Sampling. For human faeces, volunteers provided faecal samples in containers given to them. For dog faeces, in Thailand and Cambodia dogs were restrained by their owner, the dogs given a saline enema by a veterinary surgeon, then followed until the dogs defecated, when the faeces were collected. In Bangladesh, dog faeces were collected from the ground, taking care to only collect the upper part of the faecal mass.

Human and dog faecal samples were individually cultured as (1), and maintained at local ambient temperature. After 72 hours *Strongyloides* iL3s were collected, washed once in 1% w/v sodium dodecyl sulphate, twice in distilled water, then transferred individually with 10 µL of distilled water (Bangladesh and Thailand) or absolute ethanol (Cambodia) into a microcentrifuge tube; samples in water were stored at -80 ^{o}C; samples in ethanol were stored at ambient temperature.

DNA sequencing and quality control. DNA lysates were purified using AMPure XP beads, and 300 bp libraries constructed with the NEBNext Ultra II FS Kit using the one tenth, reduced volume protocol. The libraries were sequenced on the Illumina NovaSeq platform generating 2x150 bp reads. Sequencing reads were trimmed of adapter sequences using Cutadapt version 1.2.1 (2), and further trimmed using Sickle version 1.2 (3) with a minimum window quality score of 20; reads ≤15 bp after trimming were removed.

Sequencing reads were processed through Kraken2 (4), using a customised database of the default 'Standard' database with (i) four *Strongyloides* species (*S. ratti, S. papillosus, S. stercoralis, S. venezuelensis*) (5) and (ii) other parasitic nematodes (6). *S. stercoralis*-identified reads were then aligned to the *S. stercoralis* reference genome (GenBank ID: GCA 029582065.1) using mapper Bowtie2 version 2.4.5 (7) with default settings, and SAMtools version 1.9 (8) then removed unmapped reads.

Bioinformatics – nuclear genome. Using the filtered SNPs we calculated a genetic distance matrix for all pairwise combination of iL3s using filtered SNPs in TASSEL version 5.0 (9), and genetic distances were then plotted against geographical distances using the ggplot2 package in R, with the significance of the relationship evaluated with a Mantel test.

To investigate the genetic relationship between iL3s from people and dogs we calculated the pairwise fixation index (F_{ST}) values among all samples using VCFtools after (10). We made three pairwise comparisons: (i) Dog-Dog, (ii) Human-Human, (iii) Human-Dog. A box plot of these F_{ST} values among these groups was generated using the ggplot2 package in R. We also calculated the human-dog F_{ST} values in non-overlapping 50 kb windows across the genome. Additionally, to account for potential presence of identical siblings among iL3s sampled from individual hosts, we calculated the average nucleotide divergence (d_{XY}) for the same three pairwise comparisons using pixy v2.0.0 (11).

We also constructed a Neighbour Joining (NJ) tree of all the iL3s based on the pairwise genetic distance among samples using TASSEL 5, and generated bootstrap values from 1,000 replicates using R with vcfR (12) and ape (13) packages. The resulting tree file was then visualised with iTOL (14). We used Principal Component Analyses (PCA)

of the same data performed with PLINK 1.9 (15), which was processed and visualised in R, using tidyverse and ggplot2 packages.

We performed an admixture analysis using ADMIXTURE (16) for K values from 2 to 13, and determined the optimal K value by that with the lowest cross-validation error in R.

We calculated kinship coefficients between individual iL3s using KING v2.3.2 (17) to identify genetically identical siblings among IL3s sampled from individual hosts. Specifically, PLINK-generated BED files were used as the input. Two output files were produced: kin0, which contains kinship coefficients between iL3s from different hosts; and kin, which contains kinship coefficients between iL3s from the same host. Both were visualised as a heatmap using the ComplexHeatmap (18) package in R.

Bioinformatics – mitochondrial genome. We assembled mitochondrial genomes of each iL3 and combined samples using the 'consensus' function of BCFtools. For heterozygous genotypes the reference allele was selected for the consensus sequence.

We aligned mitochondrial genomes using MAFFT v7.310 (19) in two sets: (i) all the iL3 data referred to above and (ii) all other *Strongyloides* mitochondrial genomes; specifically, *S. fuelleborni* (GenBank OL505577.1), *S. papillosus* (NC 028622.1), *S. ratti* (NC 028623.1), *S. venezuelensis* (NC_028229.1), *S. vituli* (NC 066507.1) and with (i). We constructed a maximum likelihood (ML) tree using RaxML version 8.2.12 (20), using the General Time Reversible model with 500 bootstraps and visualised in iTOL.

To estimate the divergence time between human- and dog-derived *Strongyloides* from the ML data we (i) counted the number of substitutions per site since the last relevant common ancestor and (ii) used BEAST that uses a Bayesian approach to date phylogenies (21). In these calculations we assumed the *C. elegans* mitochondrial mutation rate of 1.05 x 10^{-7} per site per generation (22), and assumed two *Strongyloides* generations per year, after (23).

Supplementary Methods References

1. M. E. Viney, B. E. Matthews, D. Walliker, On the biological and biochemical nature of cloned populations of *Strongyloides ratti*. *J Helminthol* **66**, 45-52 (1992).
2. M. Martin, Cutadapt removes adapter sequences from high-throughput sequencing reads. *EMBnet j* **17**, 3 (2011).
3. Joshi, N. A, Fass, J. N, Sickle: A sliding-window, adaptive, quality-based trimming tool for FastQ files (Version 1.33). Available at https://github.com/najoshi/sickle. (2011)
4. D. E. Wood, J. Lu, B. Langmead, Improved metagenomic analysis with Kraken 2. *Genome Biol* **20**, 257 (2019).
5. V. L. Hunt *et al.*, The genomic basis of parasitism in the *Strongyloides* clade of nematodes. *Nat Genet* **48**, 299-307 (2016).
6. K. O'Brien *et al.*, Hunting for helminths: validation of short- and long-read shotgun metagenomics in parasite detection. In submission.
7. B. Langmead, S. L. Salzberg, Fast gapped-read alignment with Bowtie 2. *Nat Methods* **9**, 357-359 (2012).

8. H. Li *et al.*, The Sequence Alignment/Map format and SAMtools. *Bioinform* **25**, 2078-2079 (2009).
9. P. J. Bradbury *et al.*, TASSEL: software for association mapping of complex traits in diverse samples. *Bioinform* **23**, 2633-2635 (2007).
10. B. S. Weir, C. C. Cockerham, Estimating F-statistics for the analysis of population structure. *Evol* **38**, 1358-1370 (1984).
11. K. L. Korunes, K. Samuk, pixy: Unbiased estimation of nucleotide diversity and divergence in the presence of missing data. *Mol Ecol Resour* **21**, 1359-1368 (2021).
12. B. J. Knaus, N. J. Grünwald, vcfr: a package to manipulate and visualize variant call format data in R. *Mol Ecol Resour* **17**,44-53 (2017).
13. E. Paradis, K. Schliep, ape 5.0: an environment for modern phylogenetics and evolutionary analyses in R. *Bioinformatics* **35**,526-528 (2019).
14. Letunic, P. Bork, Interactive tree of life (iTOL) v5: an online tool for phylogenetic tree display and annotation. *Nucleic Acids Res* **49**, W293-W296 (2021).
15. S. Purcell *et al.*, PLINK: a tool set for whole-genome association and population-based linkage analyses. *Am J Hum Genet* **81**, 559-575 (2007).
16. D. H. Alexander, J. Novembre, K. Lange, Fast model-based estimation of ancestry in unrelated individuals. *Genome Res* **19**, 1655-1664 (2009).
17. A. Manichaikul *et al.* Robust relationship inference in genome-wide association studies. *Bioinform* **26**, 2867-2873 (2010).
18. Z. Gu, R. Eils, M. Schlesner, Complex heatmaps reveal patterns and correlations in multidimensional genomic data. *Bioinform* **32**, 2847-2849 (2016).
19. K. Katoh, D. M. Standley, MAFFT multiple sequence alignment software version 7: improvements in performance and usability. *Mol Biol Evol* **30**, 772-780 (2013).
20. A. Stamatakis, RAxML version 8: a tool for phylogenetic analysis and post-analysis of large phylogenies. *Bioinform* **30**, 1312-1313 (2014).
21. Bouckaert R *et al.* BEAST 2: a software platform for Bayesian evolutionary analysis. *PLoS Comput Biol* **10,** e1003537 (2014).
22. A. Konrad *et al.*, Mitochondrial mutation rate, spectrum and heteroplasmy in *Caenorhabditis elegans* spontaneous mutation accumulation lines of differing population size. *Mol Biol Evol* **34**, 1319-1334 (2017).
23. R. Cole, N. Holroyd, A. Tracey, M. Berriman, M. E. Viney, The parasitic nematode *Strongyloides ratti* exists predominantly as populations of long-lived asexual lineages. *Nat Commun* **14**, 6427 (2023).

Figure S1. Alignment of sequence reads to the *S. stercoralis* reference genome. The average read depth and genome coverage for (A) all 298 iL3s and (B) for the 143 that passed our quality control criteria. The country and host origin of samples is shown by colour and shape.

A

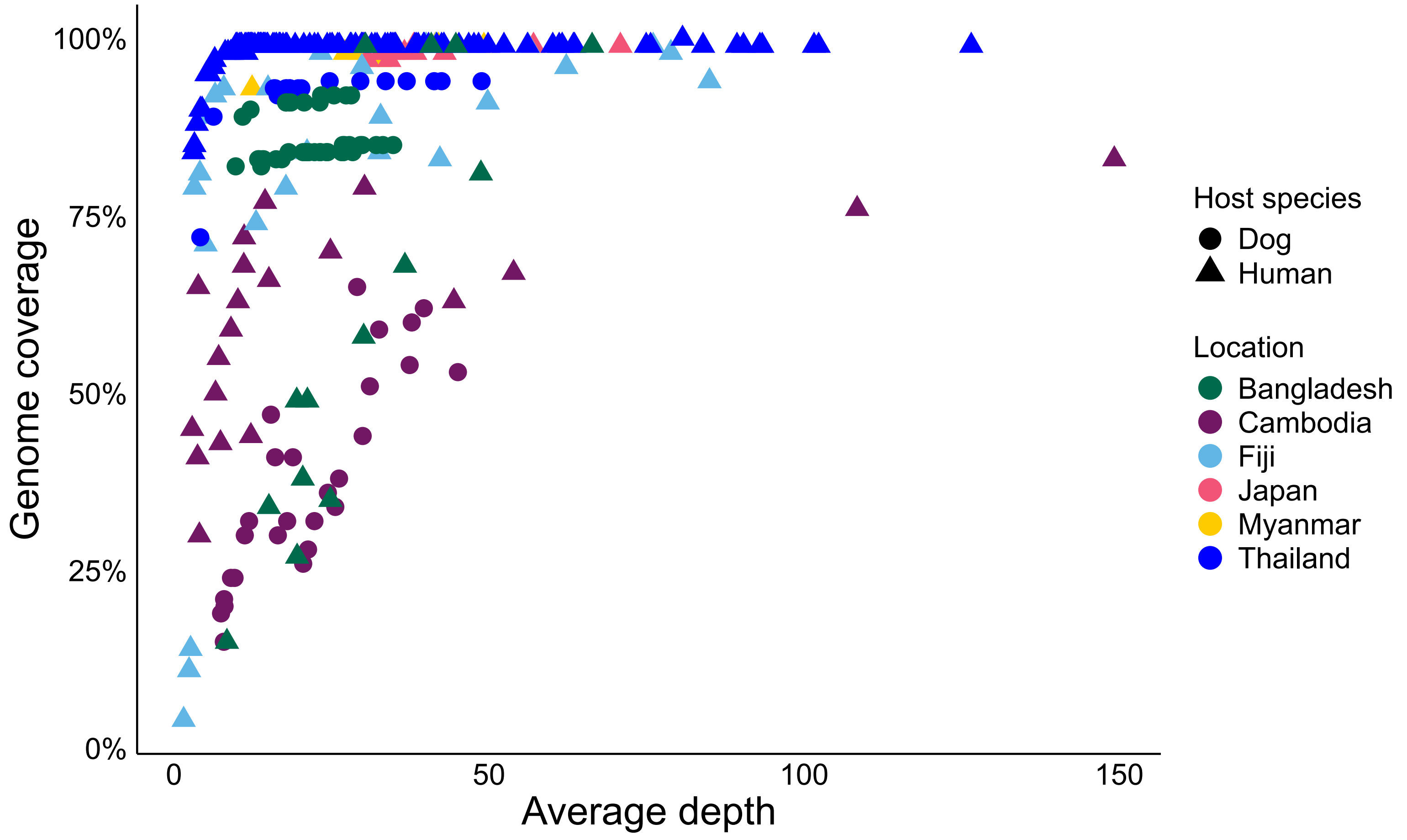

B

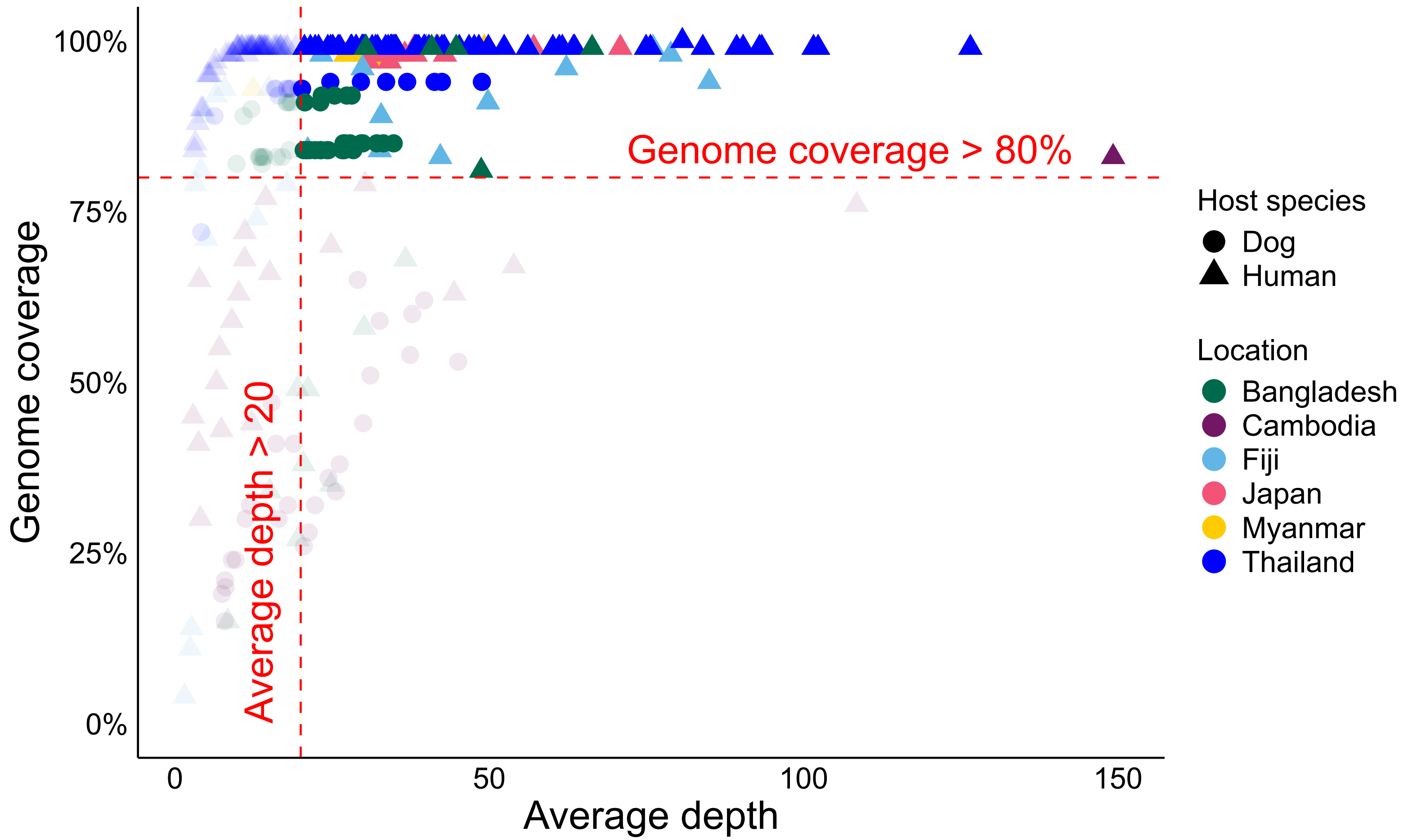

Genome coverage
100%
75%
50%
25%
0%
Average depth
0
50
100
150
Genome coverage > 80%
Average depth > 20
Host species
Dog
Human
Location
Bangladesh
Cambodia
Fiji
Japan
Myanmar
Thailand

Figure S2. Combined sequence samples. (A) Neighbour joining tree of 56 iL3s where the combination strategy was used. The details in areas **B, C, D** marked by dotted boxes in (A) are shown in (B), (C) and (D), respectively. Samples derived from the same host and within the same nodes are outlined by the blue curves. The scales are 1 substitution per 10 bases in (A) and (D), 1 substitution per 100 bases in (B), and 1 substitution per 1000 bases in (C).

A

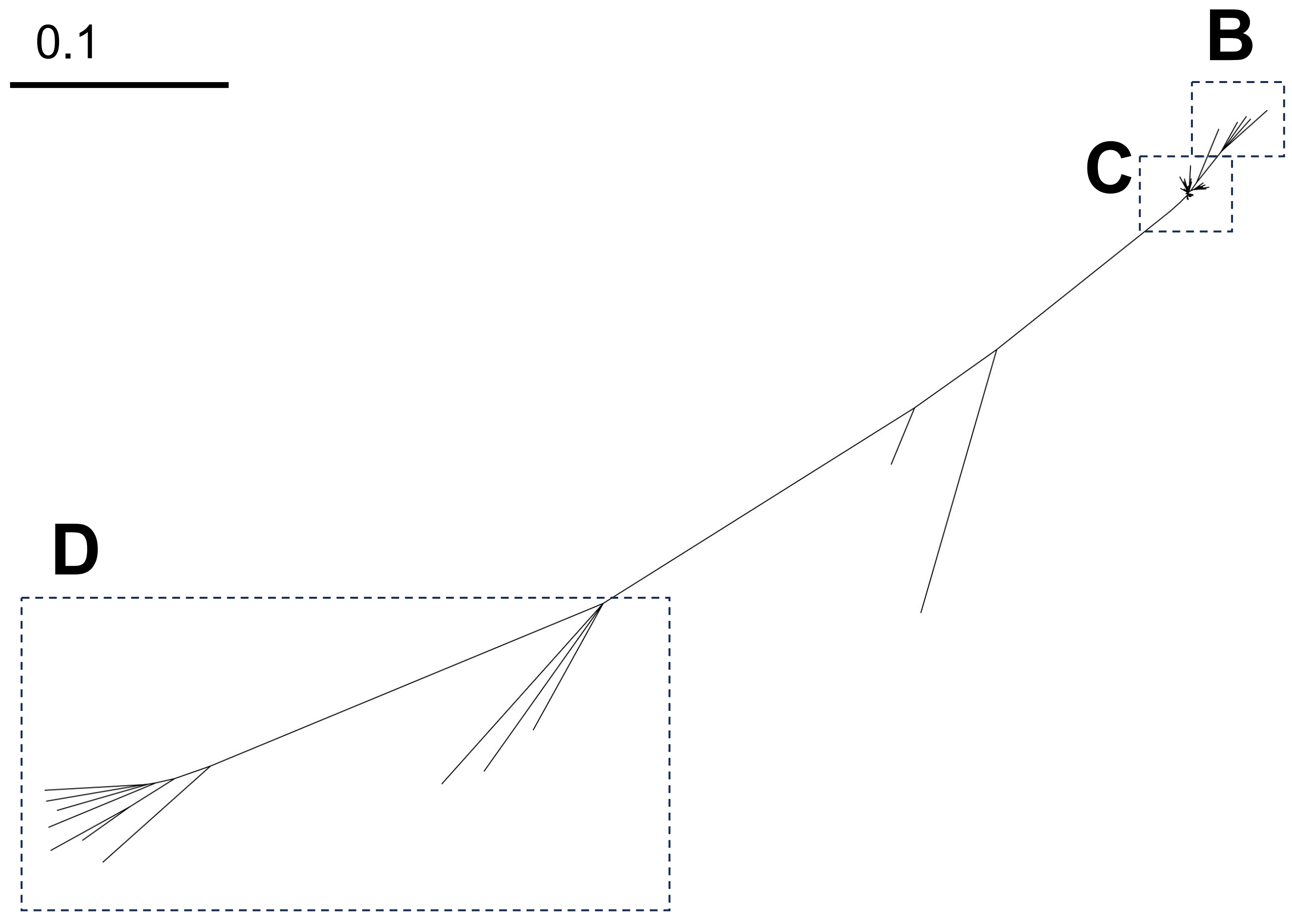

B

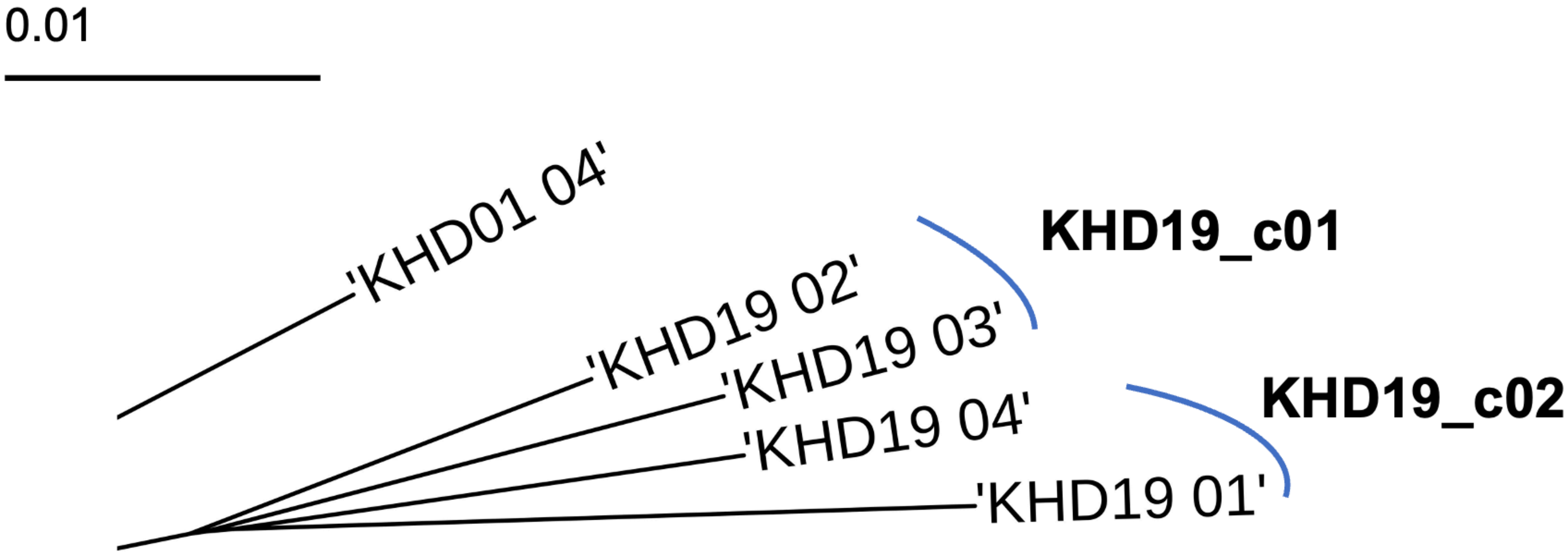
0.01
'KHD01 04'
'KHD19 02'
'KHD19 03'
'KHD19 04'
'KHD19 01'
KHD19_c01
KHD19_c02

c

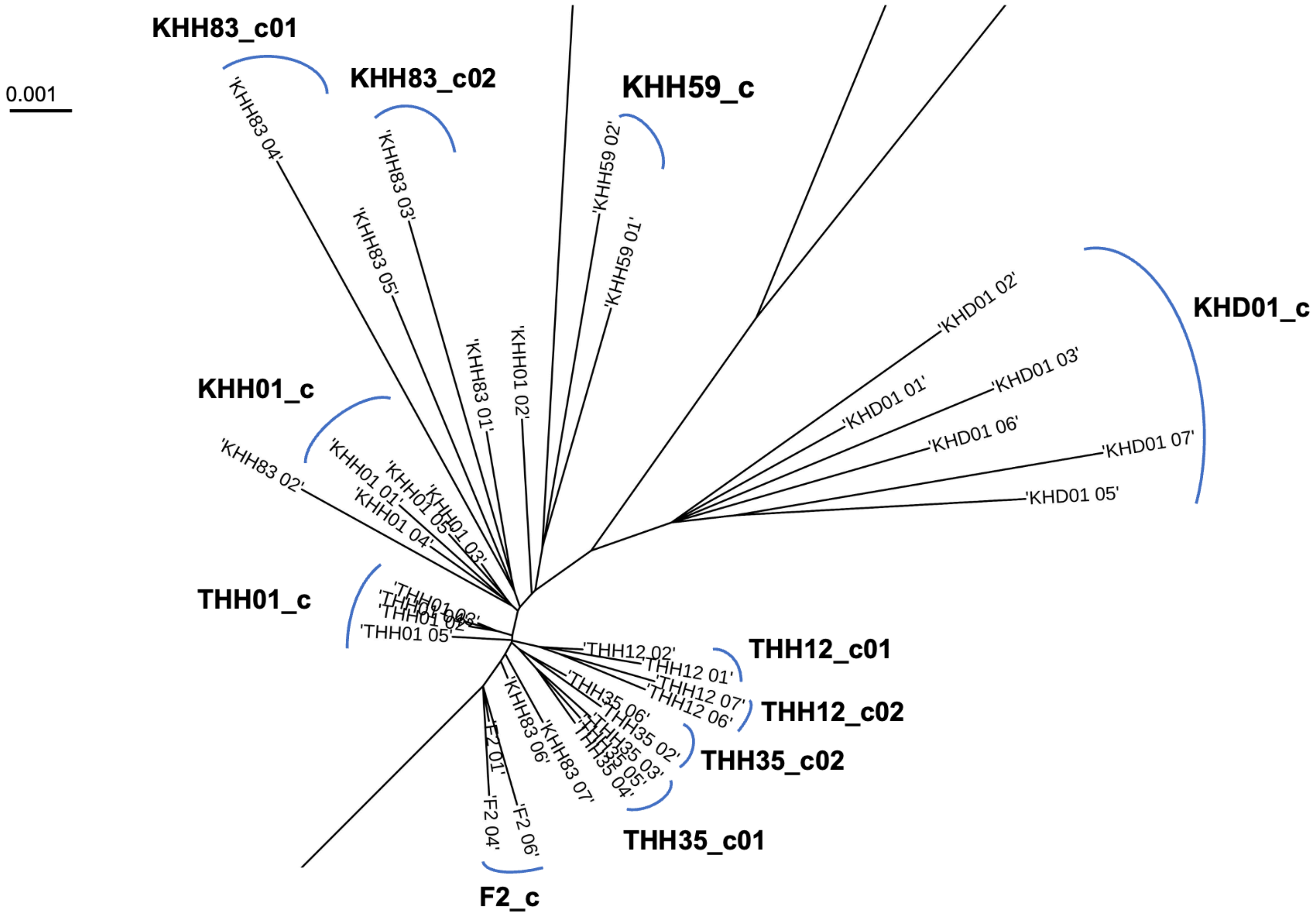
0.001
KHH83_c01
KHH83_c02
KHH59_c
KHD01_c
KHH01_c
THH01_c
THH12_c01
THH12_c02
THH35_c02
THH35_c01
F2_c
'KHH83 04'
'KHH83 03'
'KHH83 05'
'KHH83 01'
'KHH01 02'
'KHH59 02'
'KHH59 01'
'KHD01 02'
'KHD01 03'
'KHD01 01'
'KHD01 06'
'KHD01 07'
'KHD01 05'
'KHH83 02'
'KHH01 01'
'KHH01 04'
'KHH01 03'
'THH01 05'
'THH12 02'
'THH12 01'
'THH12 07'
'THH12 06'
'THH35 06'
'THH35 02'
'THH35 03'
'THH35 04'
'KHH83 06'
'KHH83 07'
'F2 01'
'F2 04'
'F2 06'

D

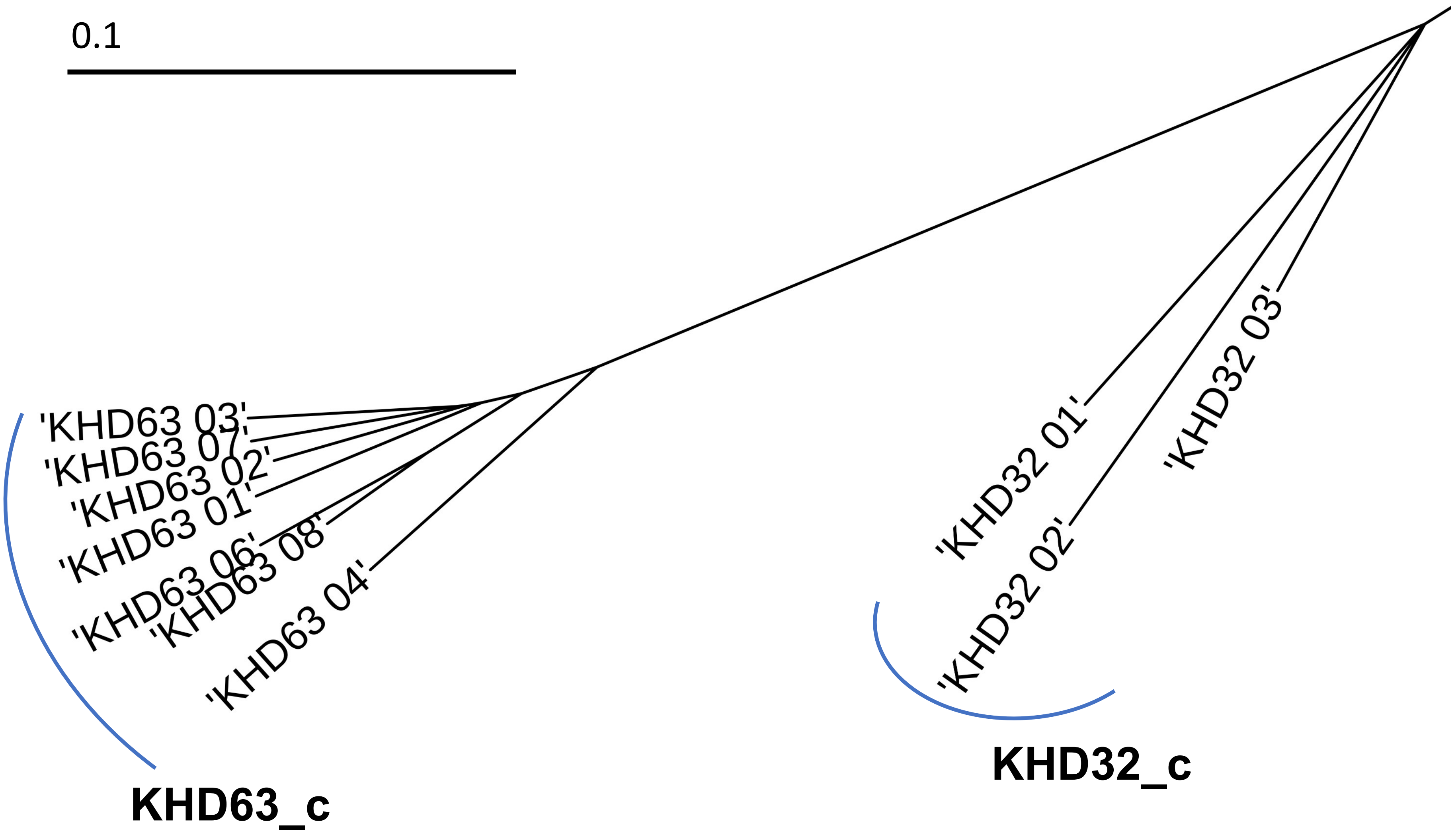
0.1
'KHD63 03'
'KHD63 07'
'KHD63 02'
'KHD63 01'
'KHD63 06'
'KHD63 08'
'KHD63 04'
KHD63_c
'KHD32 01'
'KHD32 02'
'KHD32 03'
KHD32_c

Figure S3. Principal Component Analysis. Plots of Principal Components (PCs) 1 and 2 of (A) all 159 iL3s, (B) those within cluster D, (C) those within cluster D2. The iL3s are shaped and coloured by host species and geographical location. The iL3s are grouped by clusters A, B, C, D, D1 and D2, which are as main text **Figure 2B**. PCs 1 and 2 account for 45.3 and 27.1 % of the variance, respectively. The grey and black dots in (B) indicates the *S. stercoralis* reference PV001 and 3 putative introgressed genotypes, respectively; the yellow diamond in (C) indicates the putative human-to-dog cross infection.

A

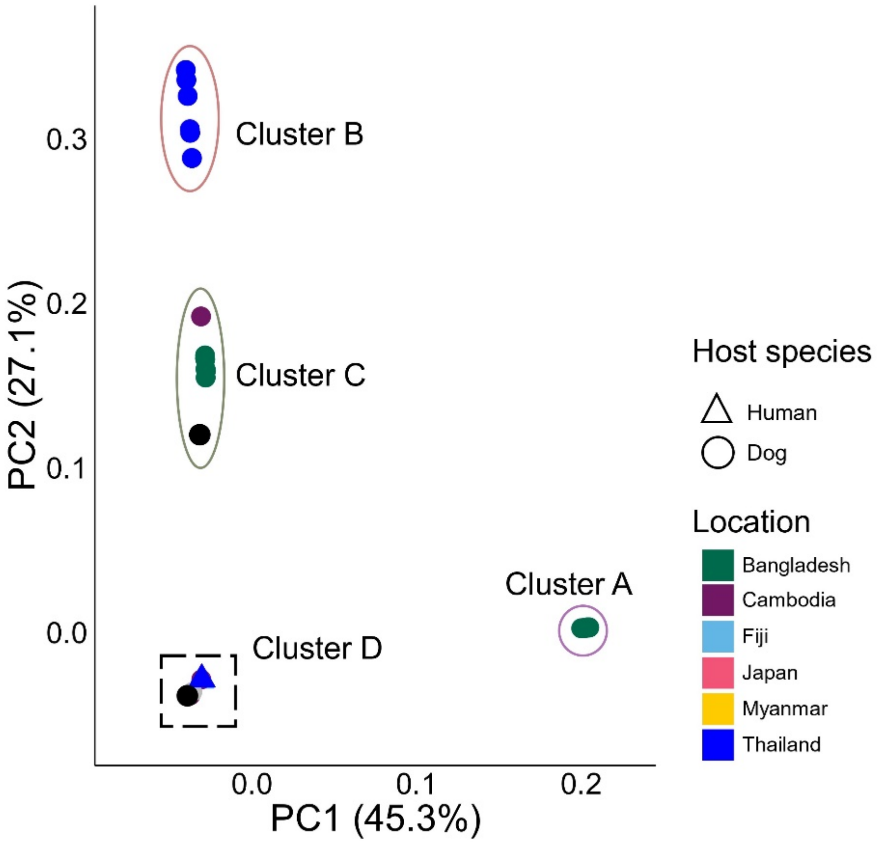

B

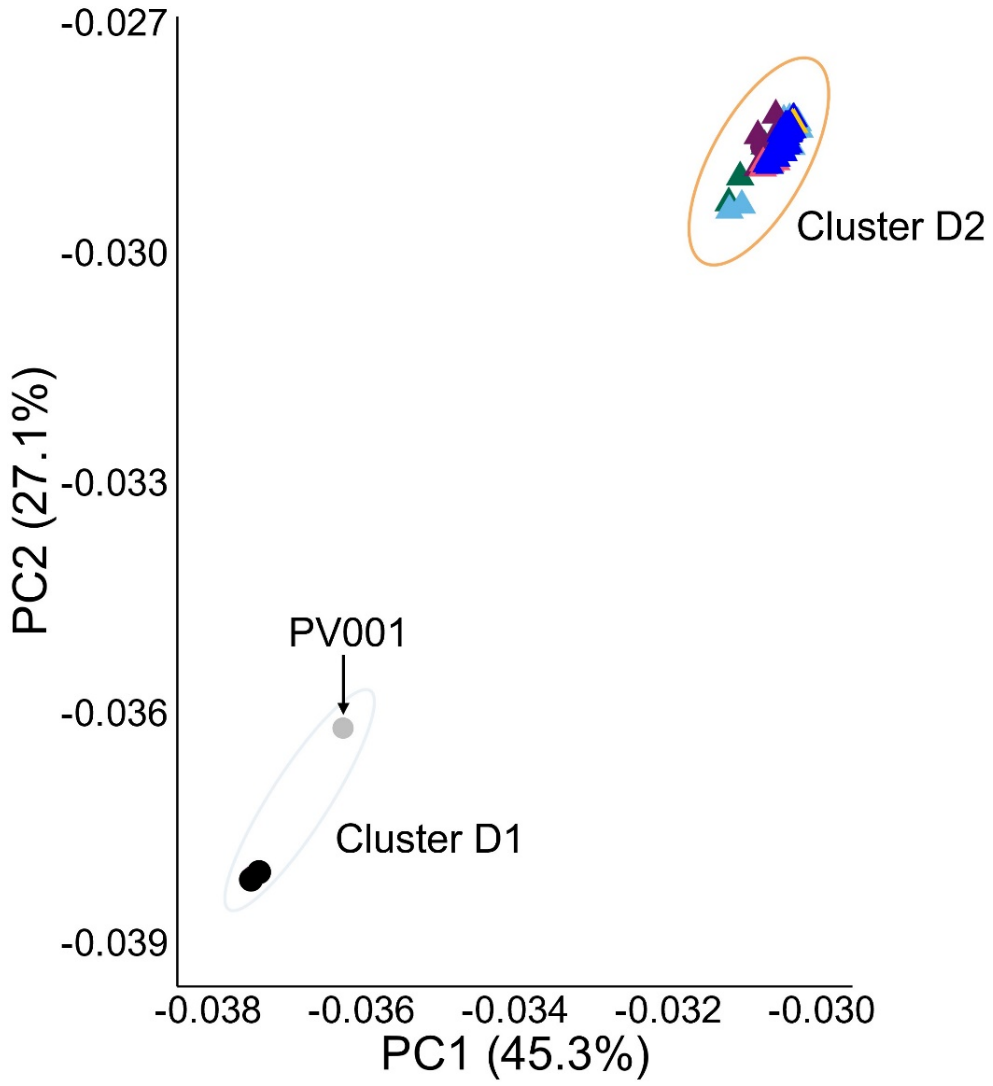

-0.027
-0.030
-0.033
-0.036
-0.039
PC2 (27.1%)
Cluster D2
PV001
Cluster D1
-0.038
-0.036
-0.034
-0.032
-0.030
PC1 (45.3%)

c

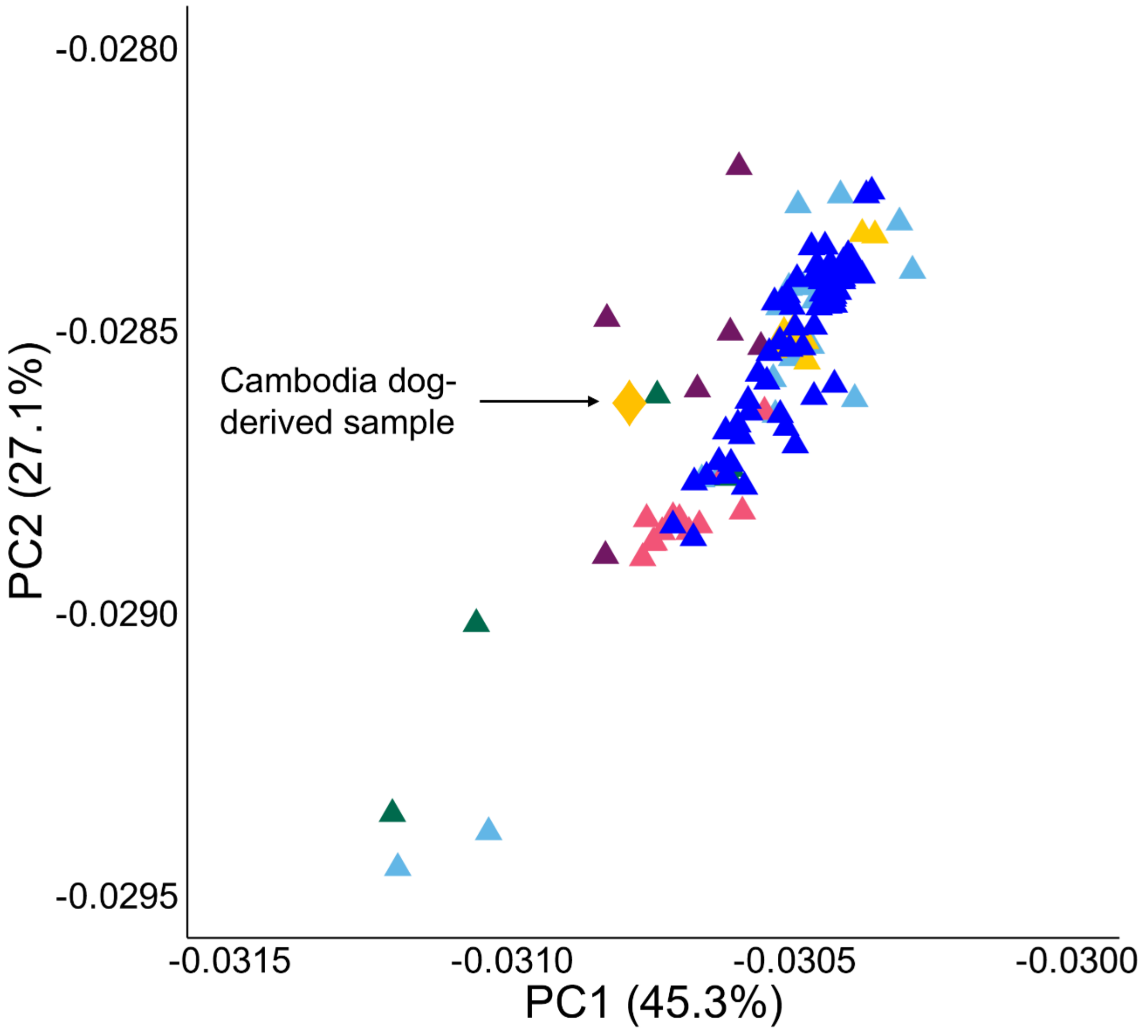

-0.0280
-0.0285
-0.0290
-0.0295
PC2 (27.1%)
Cambodia dog-
derived sample
-0.0315
-0.0310
-0.0305
-0.0300
PC1 (45.3%)

Figure S4. Distribution of genotypes among hosts. Details of cluster D1 and D2 of the neighbour joining tree in main text **Figure 2B**, showing (i) different genotypes co-infecting a host (blue boxes) and (ii) different hosts in which closely related genotypes occur (red boxes).

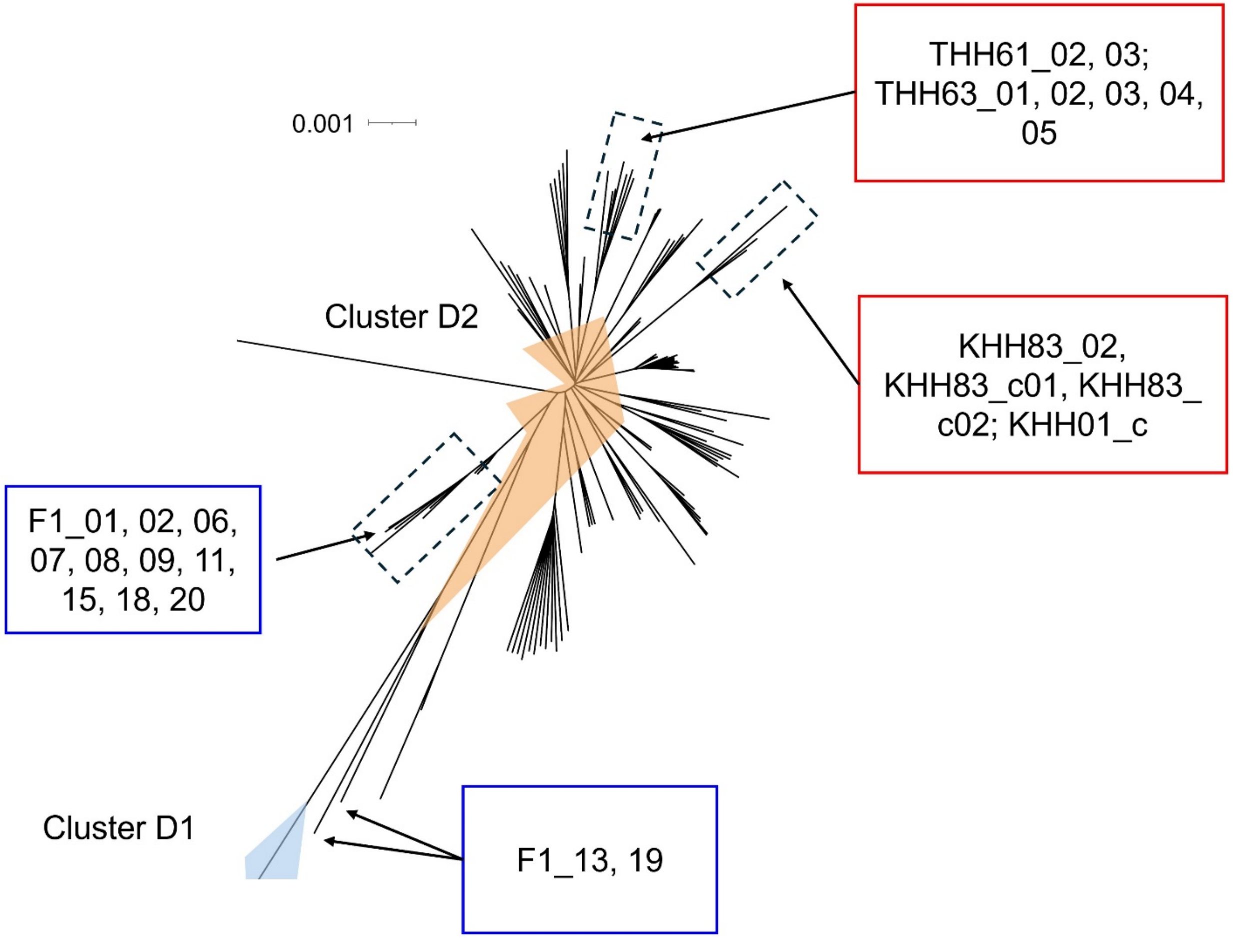

Figure S5. Admixture. (A) Admixture for k = 5. The proportion of admixture and the distribution of genetic ancestries for each iL3, with host species and geographical location of iL3s shown at the top of the plot, and the nuclear clusters of each at the bottom. (B) Admixture for k = 8, as main text Figure 2C, but showing the individual iL3 identifiers.

A

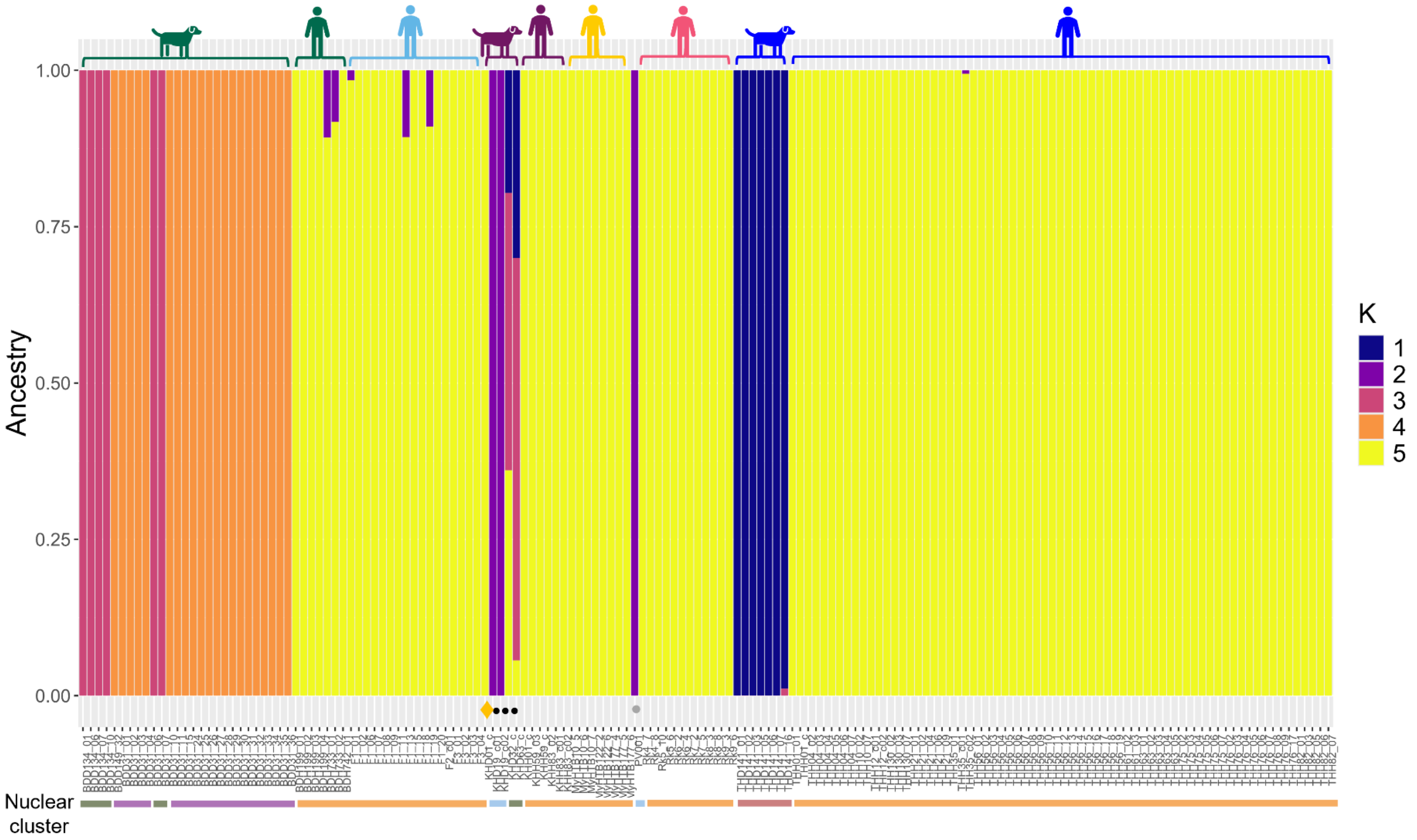

B

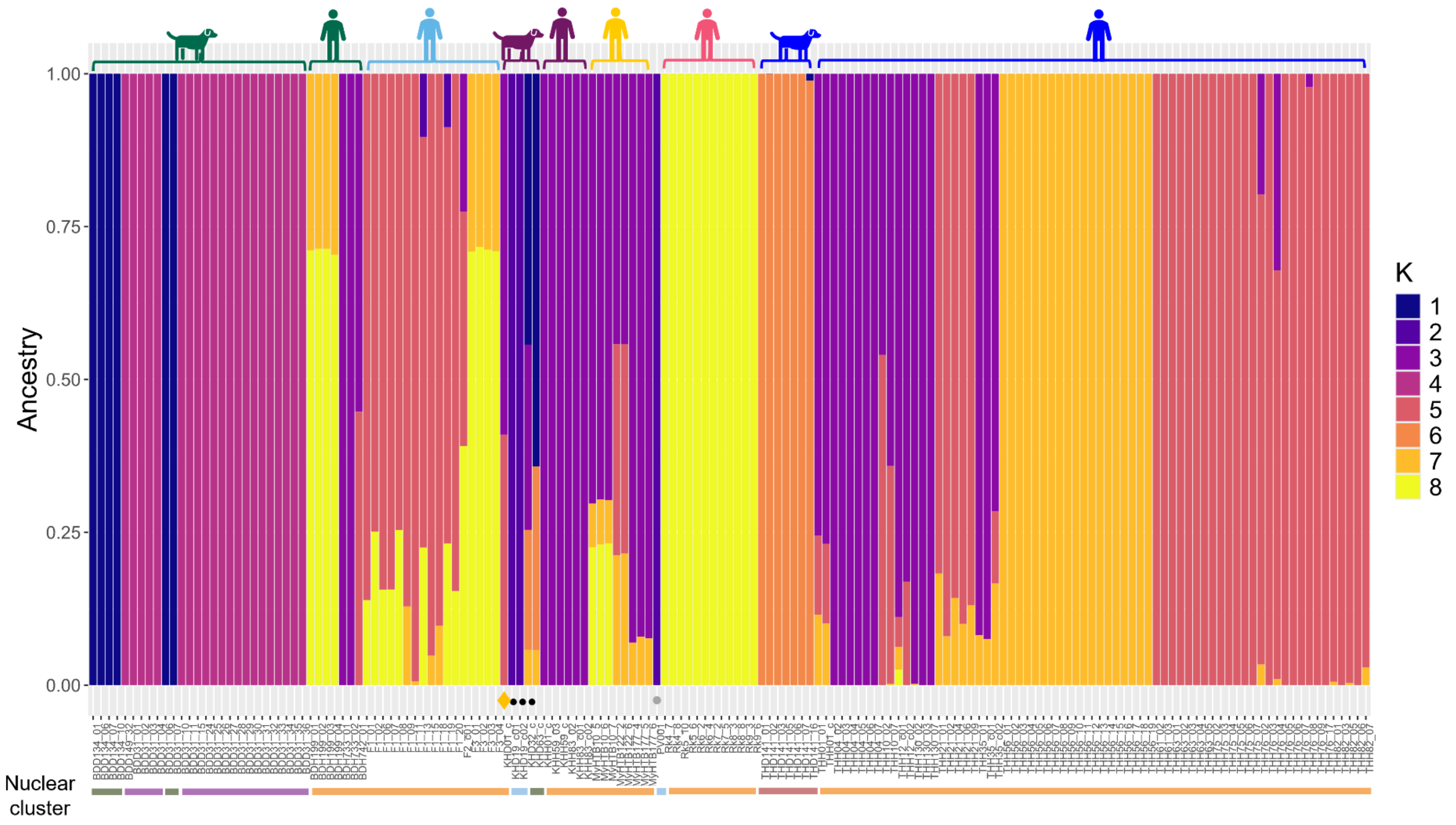

Figure S6. Nuclear neighbour joining (NJ) tree structure and admixture with sample reduction. (A) removal of the 15 combined samples leaving 144 iL3 and (B) also removing all but one iL3 from each host, leaving 34 iL3 samples. Comparison of these with the full tree (**Figure 2B**) shows that the overall structure of the trees, and the existence of the clusters A, B, C, D1 (all from dogs) and D2 (from people), is maintained. In A and B the scales are 1 substitution per 10 bases, and per 1,000 bases. (C) Admixture after removal of the 15 combined samples leaving 144 iL3s, with ADMIXTURE best supporting k = 11 groups, where 32 % (11 of 34) dog-derived samples have evidence of admixture compared to 19 % (21 of 110) for the human-derived samples. Host species and geographical location of iL3s shown at the top of the plot, and the nuclear clusters of each at the bottom. The grey dot in (A), (B) and (C) indicates the *S. stercoralis* reference PV001.

A

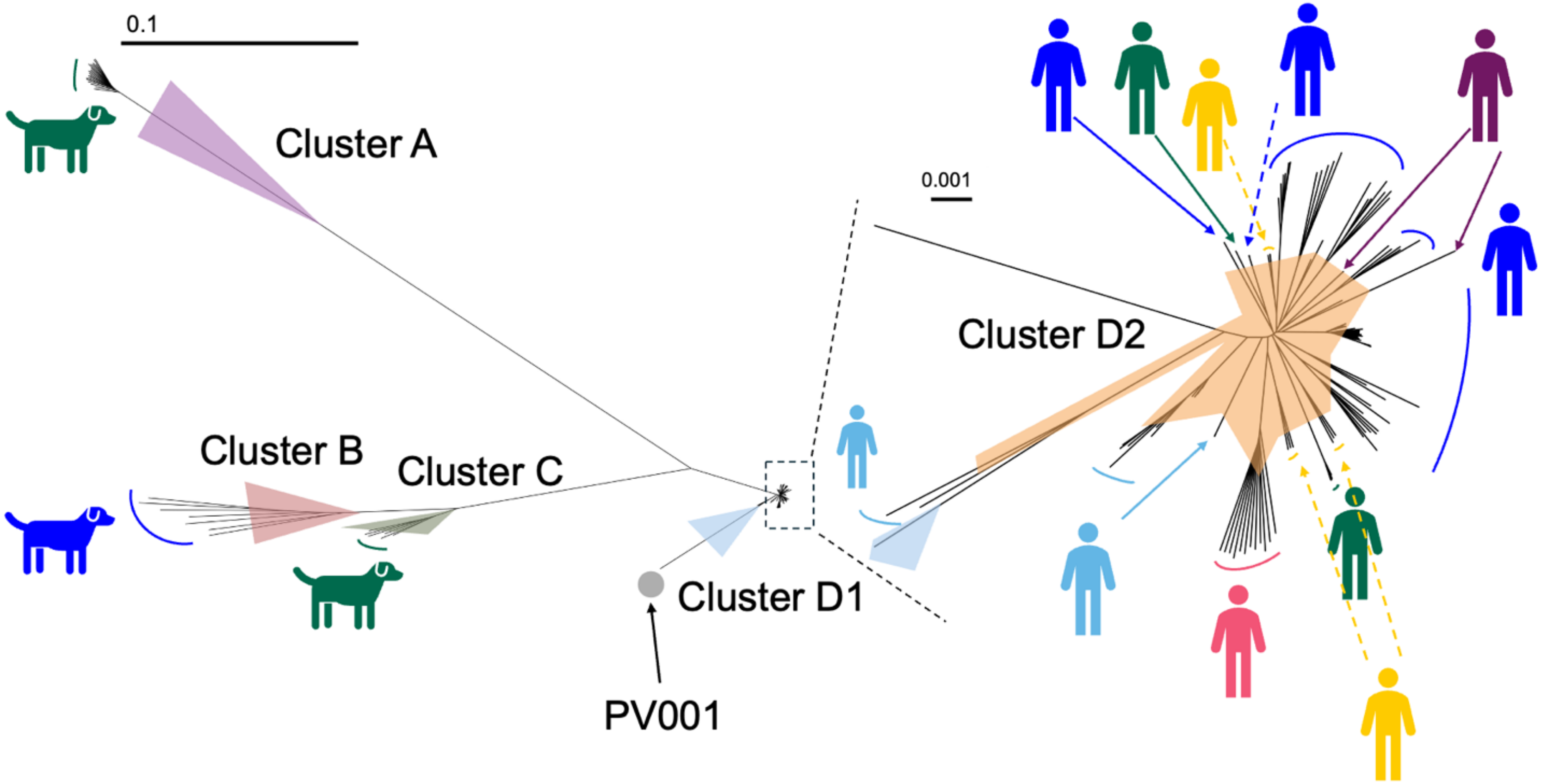

B

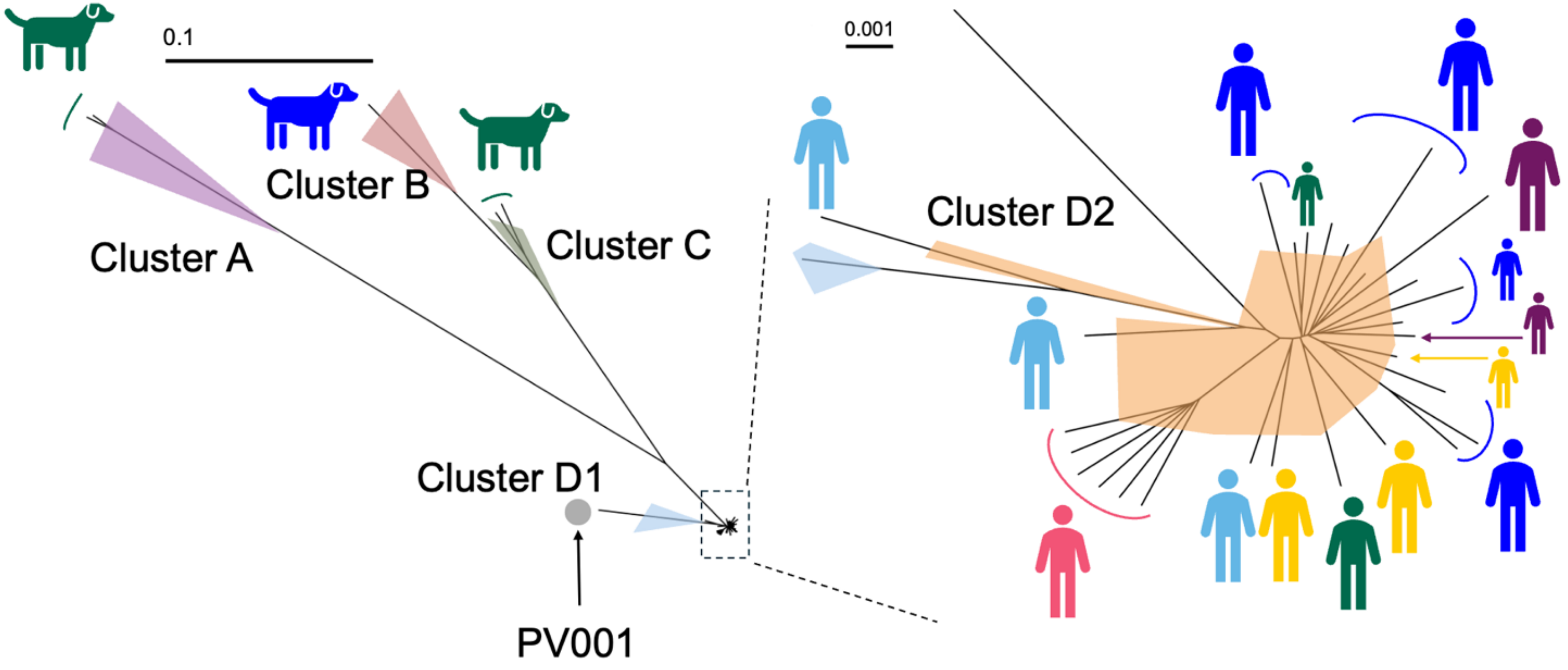
0.1
Cluster A
Cluster B
Cluster C
Cluster D1
PV001
0.001
Cluster D2

c

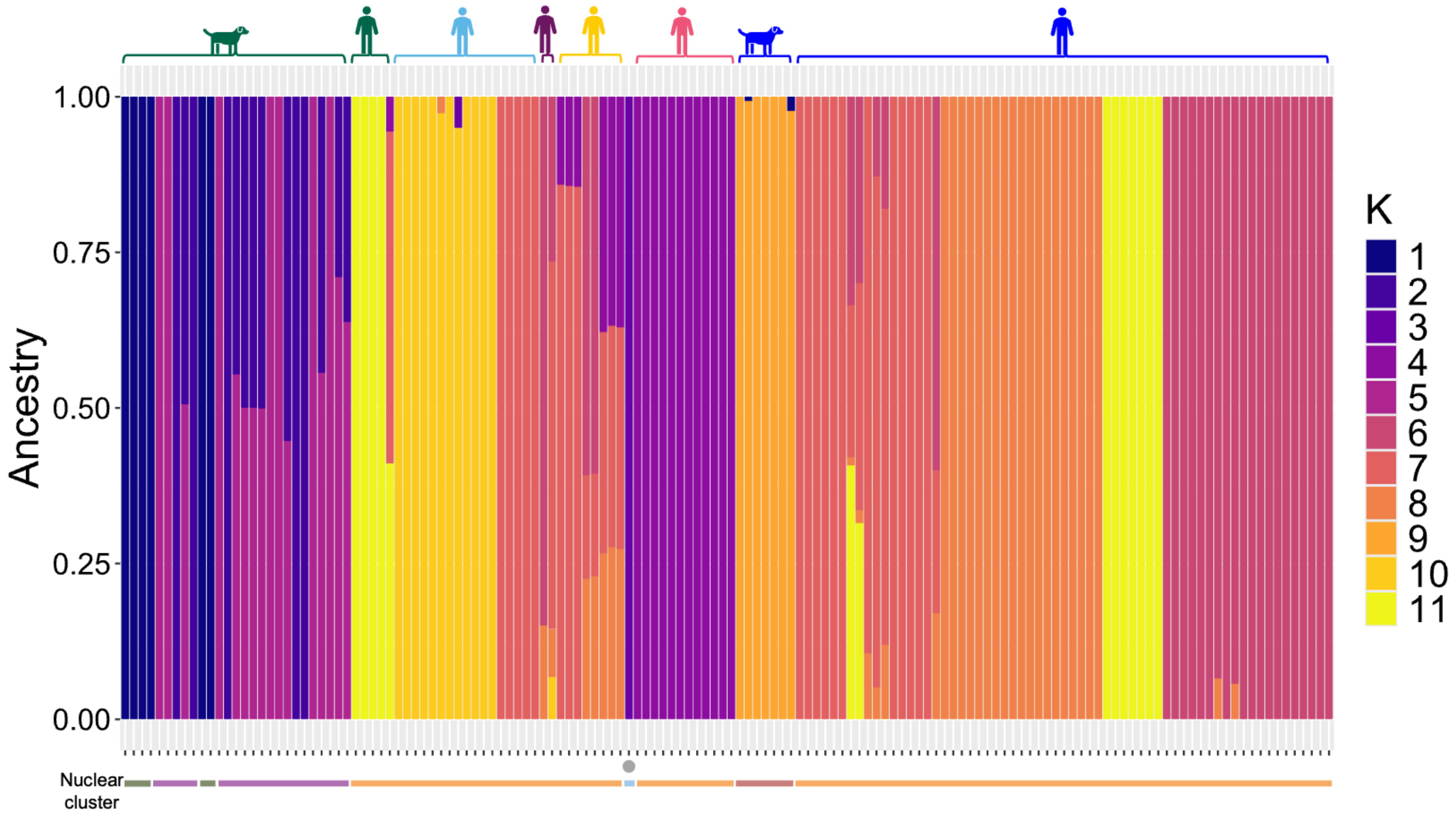
Ancestry
1.00
0.75
0.50
0.25
0.00
K
1
2
3
4
5
6
7
8
9
10
11
Nuclear cluster

Figure S7. F_{ST} values across the genome. F_{ST} values in 50 kb sliding windows across the genome among iL3s from the two different host species, humans and dogs. The x-axis shows the different chromosomes of the *S. stercoralis* genome, with 6 scaffolds of the X chromosome. Grey dashed lines show the boundaries between chromosomes and scaffolds.

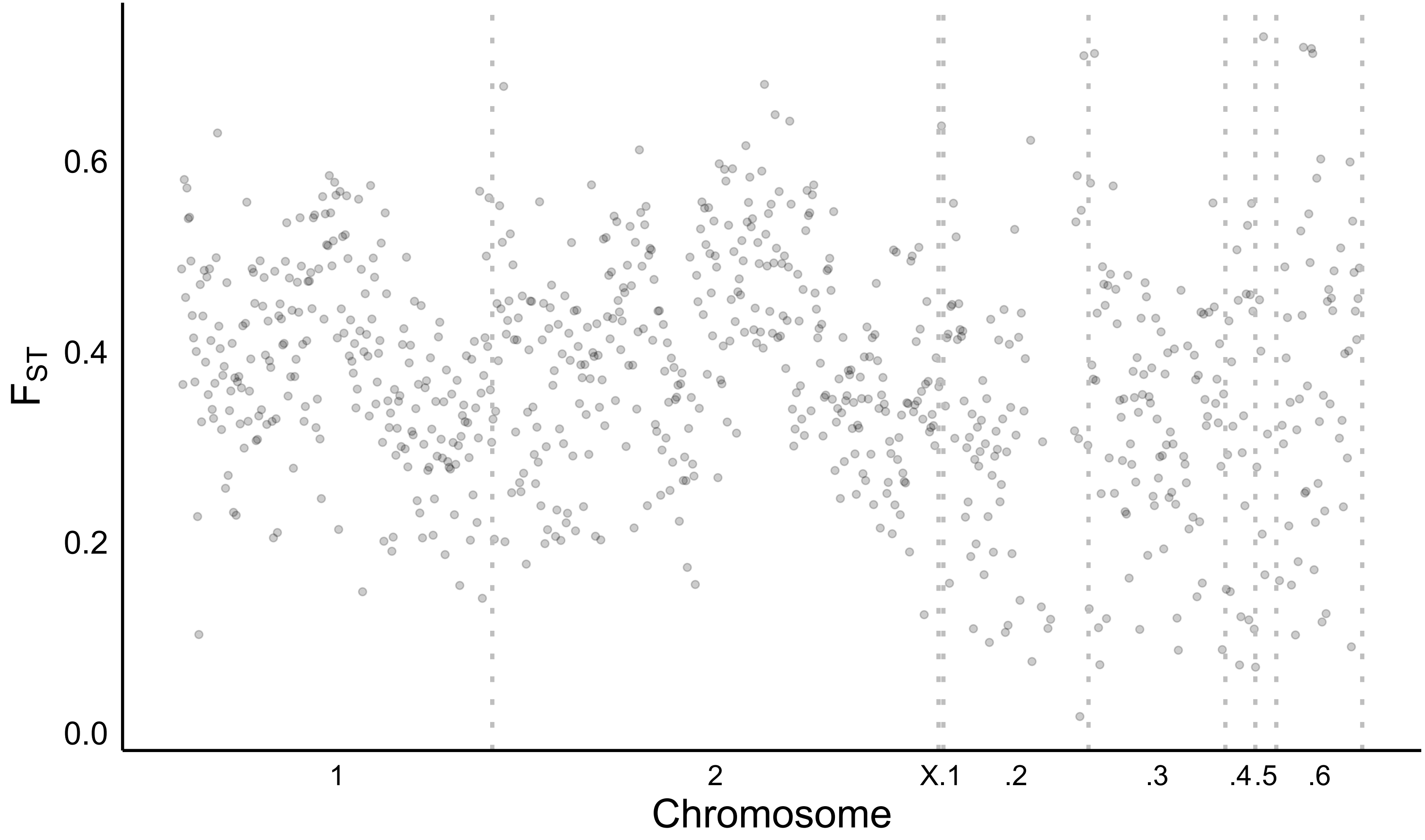

Figure S8. Trees with individual sample identifiers. (A) A neighbour joining tree of human and dog-derived iL3s, as main text **Figure 2B**, but showing the individual iL3 identifiers. (B) A maximum likelihood tree of human and dog-derived iL3s, as main text **Figure 3A**, but showing the individual iL3 identifiers. Scales are 1 substitution per 10 bases in (A), and 1 substitution per 100 bases in (B).

A

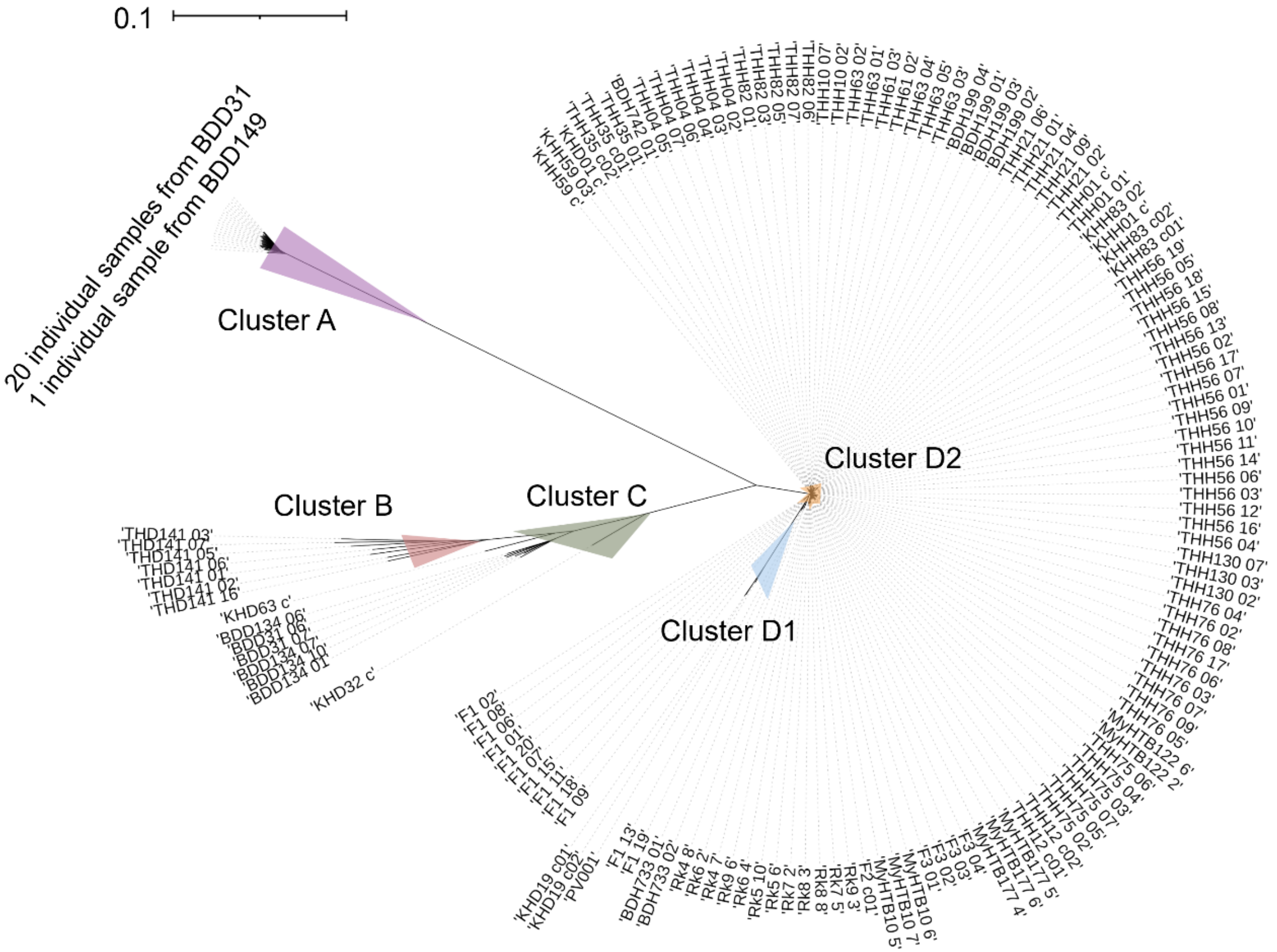

B

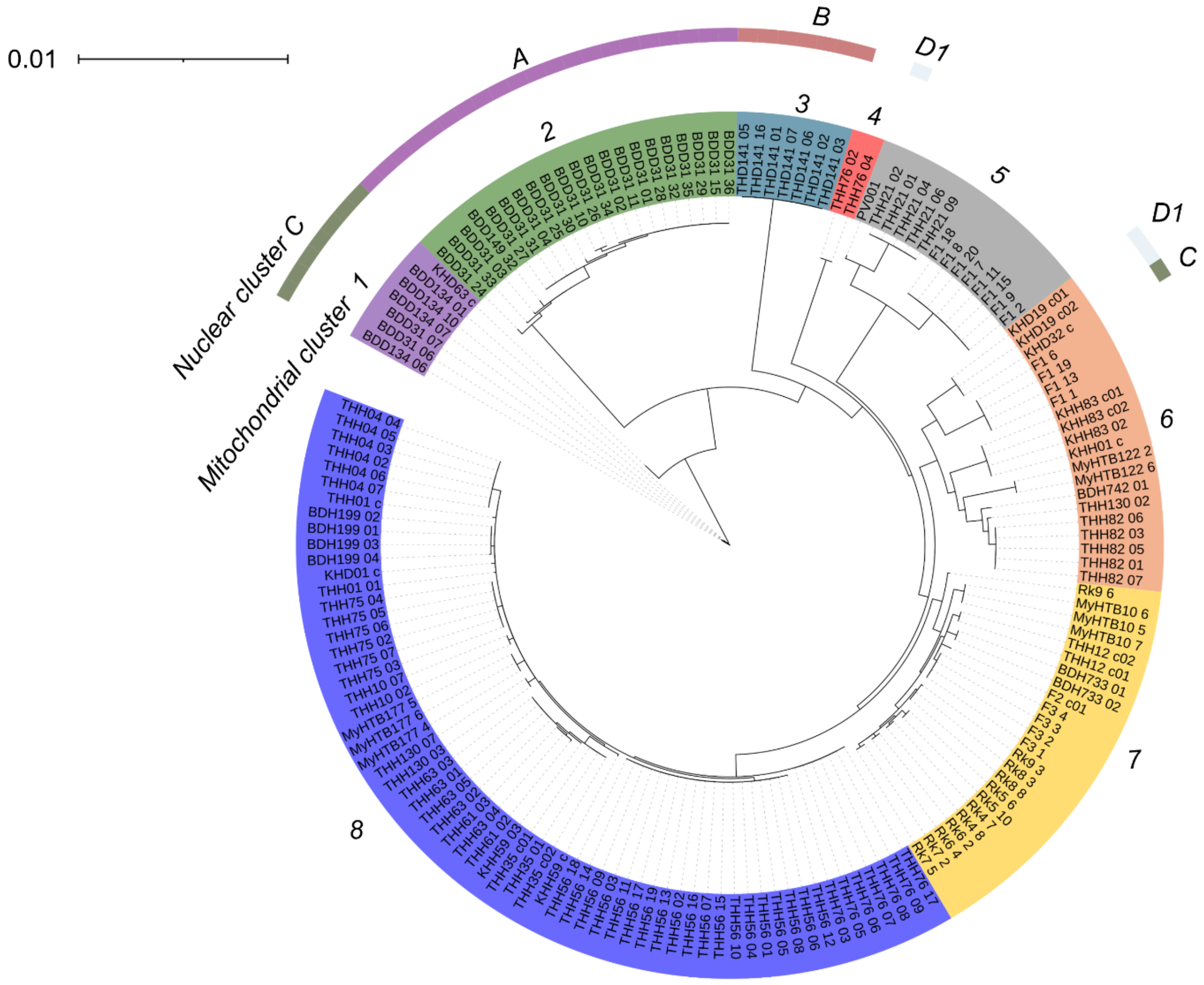
0.01
A
B
D1
D1
C
Nuclear cluster C
Mitochondrial cluster 1
2
3
4
5
6
7
8
KHD63 c
BDD134 01
BDD134 10
BDD134 07
BDD31 07
BDD31 06
BDD134 06
BDD31 36
BDD31 15
BDD31 29
BDD31 35
BDD31 32
BDD31 28
BDD31 11
BDD31 01
BDD31 02
BDD31 34
BDD31 26
BDD31 10
BDD31 30
BDD31 25
BDD31 04
BDD31 37
BDD31 27
BDD31 24
THD141 05
THD141 16
THD141 01
THD141 07
THD141 06
THD141 02
THD141 03
THH76 02
THH76 04
PV001
THH21 02
THH21 01
THH21 04
THH21 06
THH21 09
F1 18
F1 20
KHD19 c01
KHD19 c02
KHD32 c
F1 6
F1 19
F1 13
F1 1
KHH83 c01
KHH83 c02
KHH83 02
KHH01 c
MyHTB122 2
MyHTB122 6
BDH742 01
THH130 02
THH82 06
THH82 03
THH82 05
THH82 01
THH82 07
Rk9 6
MyHTB10 6
MyHTB10 5
MyHTB10 7
THH12 c02
THH12 c01
BDH733 01
BDH733 02
THH76 17
THH76 09
THH76 08
THH76 07
THH76 06
THH76 05
THH76 03
THH56 12
THH56 06
THH56 08
THH56 05
THH56 01
THH56 04
THH56 10
THH56 15
THH56 07
THH56 16
THH56 02
THH56 13
THH56 19
THH56 17
THH56 11
THH56 03
THH56 09
THH56 14
THH56 18
KHH59 c
THH35 c02
THH35 c01
KHH59 03
THH63 03
THH130 03
THH130 07
MyHTB177 4
MyHTB177 6
MyHTB177 5
THH10 02
THH10 07
THH75 03
THH75 07
THH75 02
THH75 06
THH75 05
THH75 04
THH01 01
KHD01 c
BDH199 04
BDH199 03
BDH199 01
BDH199 02
THH01 c
THH04 07
THH04 06
THH04 02
THH04 03
THH04 05
THH04 04

Table S1. Origins of samples. (A) Location of samples sites in Bangladesh, Thailand and Cambodia. (B) Number of human and dog faecal samples, number of *Strongyloides* positives, and *Strongyloides* prevalence. (C) The number of iL3s collected and successfully sequenced from individual hosts. Failed library is where no *Strongyloides* sequence data were generated; Identified as *Strongyloides* is the Kraken-defined *Strongyloides* species identity.

A

Site	Coordinates	Rural *vs*. urban classification
**Bangladesh**		
BD site 1	22°19'41"N 91°50'24"E	Suburb
BD site 2	22°19'55"N 91°51'24"E	Suburb
BD site 3	22°19'29"N 91°49'14"E	Suburb
BD site 4	22°21'37.5"N 91°47'19"E	Urban
BD site 5	22°22'22"N 91°50'07"E	Urban
**Thailand**		
TH site 1	16°07'53"N 102°39'01"E	Rural
TH site 2	16°09'52"N 102°39'49"E	Rural
TH site 3	16°05'56"N 102°41'01"E	Rural
TH site 4	16°18'29"N 102°38'35"E	Rural
TH site 5	16°16'40"N 102°39'21"E	Rural
TH site 6	16°28'11"N 102°49'52"E	Hospital (one sample)
**Cambodia**		
Village in Rovieng	13°22'01"N 105°06'57"E	Rural

B

Site	No. human samples	No. positive	Human *Strongyloides* prevalence (95% CI)	No. dog samples	No. positive	Dog *Strongyloides* prevalence (95% CI)
**Bangladesh**						
BD site 1	93	2	2.2% (0.3 – 7.6%)	45	2	4.4% (0.5– 15.2%)
BD site 2	83	1	1.2% (0.3 – 6.5%)	75	1	1.3% (0.3 – 7.2%)
BD site 3	14	0	0	20	0	0
BD site 4	9	0	0	30	0	0
BD site 5	12	0	0	14	0	0
**Sum**	**211**	**3**	**1.4% (0.3 – 4.1%)**	**184**	**3**	**1.6% (0.3 – 4.7%)**
**Thailand**						
TH site 1	121	10	8.3% (4 – 14.7%)	43	0	0
TH site 2	34	2	5.9% (0.7 – 19.7%)	37	0	0
TH site 3	0	0	0	16	0	0
TH site 4	0	0	0	25	0	0
TH site 5	0	0	0	18	1	5.6% (0.1 – 27.3%)
TH site 6	1	1		0	0	
**Sum (except site 6)**	**155**	**12**	**7.7% (4.1 – 13.1%)**	**139**	**1**	**0.7% (0.2 – 3.9%)**
**Cambodia**						
**Village in Roveing**	**102**	**8**	**7.8% (3.5 – 14.9%)**	**71**	**6**	**8.5% (3.2 – 17.5%)**
**Overall**	**468**	**23**	**4.9% (3 – 7.3%)**	**394**	**10**	**2.5% (1.2 – 4.6%)**

C

			Number of iL3s					
						Identified as *Strongyloides*		
**Country**	**Host**	**Host ID**	**Collected**	**Sequenced**	**Failed library**	***stercoralis***	***ratti***	***venezuelensis***
**Bangladesh**	Human	BDH199	4	4	0	4	0	0
		BDH733	36	5	0	5	0	0
		BDH742	>250	5	0	5	0	0
	Dog	BDD31	38	36	4	32	0	0
		BDD134	17	12	2	10	0	0
		BDD149	78	43	6	1	2	34
	**Total**	**3 people + 3 dogs**		**105**	**12**	**57**	**2**	**34**
**Cambodia**	Human	KHH01	20	5	0	5	0	0
		KHH43	1	1	0	1	0	0
		KHH54	27	1	0	1	0	0
		KHH59	10	3	0	3	0	0
		KHH76	10	3	0	3	0	0
		KHH83	50	7	0	7	0	0
	Dog	KHD01	35	7	0	7	0	0
		KHD15	10	3	0	3	0	0
		KHD19	10	4	0	4	0	0
		KHD27	10	2	0	2	0	0
		KHD32	10	3	0	3	0	0
		KHD63	61	8	0	8	0	0
	**Total**	**6 people + 6 dogs**		**47**	**0**	**47**	**0**	**0**
**Thailand**	Human	THH01	53	7	0	7	0	0
		THH04	18	7	0	7	0	0
		THH10	52	7	0	7	0	0
		THH12	19	7	0	7	0	0

		THH21	>1000	20	0	20	0	0
		THH35	40	7	0	7	0	0
		THH56	>1000	20	0	20	0	0
		THH61	3	3	0	3	0	0
		THH63	30	7	0	7	0	0
		THH75	50	7	1	6	0	0
		THH76	>500	19	0	19	0	0
		THH82	15	8	0	8	0	0
		THH130	45	7	0	7	0	0
	Dog	THD141	18	18	0	18	0	0
	**Total**	**13 people + 1 dog**		**144**	**1**	**143**	**0**	**0**
**Fiji**	Human	F1	82	20	1	19	0	0
		F2	20	6	0	6	0	0
		F3	10	4	0	4	0	0
	**Total**	**3 people**		**30**	**1**	**29**	**0**	**0**
**All samples collected in the present study**	**Human**	**25 people**		**190**	**2**	**188**	**0**	**0**
	**Dog**	**10 dogs**		**136**	**12**	**88**	**2**	**34**
	**Total**	**25 people + 10 dogs**		**326**	**14**	**276**	**2**	**34**

Table S2. Combined sequence samples. The average depth and genome coverage of 56 iL3s, and then combined (shown in bold). The combined samples are named with the suffix “c” with the contributing individual host IDs in parentheses.

Sample ID	Average depth	Genome coverage
KHH01 01	4	41%
KHH01 02	7	55%
KHH01 03	11	68%
KHH01 04	9	59%
KHH01 05	25	70%
**KHH01_c(03-05)**	**52**	**97%**
KHH59 01	11	72%
KHH59 02	10	63%
**KHH59_c(01-02)**	**21**	**88%**
KHH83 01	14	77%
KHH83 03	108	76%
KHH83 04	44	63%
KHH83 05	54	67%
KHH83 06	3	45%
KHH83 07	4	65%
**KHH83_c01 (01and03)**	**123**	**94%**
**KHH83_c02 (04and05)**	**98**	**87%**
KHD01 01	18	32%
KHD01 02	26	34%
KHD01 03	45	53%
KHD01 04	8	20%
KHD01 05	16	41%
KHD01 06	9	24%
KHD01 07	10	24%
**KHD01_c(01,02,03,05,06,07)**	**114**	**89%**
KHD19 01	33	59%
KHD19 02	15	47%
KHD19 03	29	65%
KHD19 04	38	60%
**KHD19_c01 (02and03)**	**44**	**81%**
**KHD19_c02 (01and04)**	**70**	**83%**
KHD32 01	31	51%
KHD32 02	40	62%
KHD32 03	19	41%
**KHD32(c(01-03)**	**90**	**88%**
KHD63 01	22	32%
KHD63 02	21	26%
KHD63 03	30	44%

KHD63 04	16	30%
KHD63 05	12	32%
KHD63 06	24	36%
KHD63 07	21	28%
KHD63 08	8	15%
**KHD63_c(1,2,3,4,6,7,8)**	**153**	**89%**
F2 01	7	92%
F2 02	3	79%
F2 03	4	81%
F2 04	8	93%
F2 05	5	89%
F2 06	17	99%
**F2_c01(1,4,6)**	**31**	**99%**
THH01 02	16	99%
THH01 03	12	98%
THH01 04	13	99%
THH01 05	13	99%
**THH01_c(2-5)**	**53**	**99%**
THH12 01	12	99%
THH12 02	15	99%
THH12 03	10	98%
THH12 06	12	99%
THH12 07	14	99%
**THH12_c01 (1,2)**	**26**	**99%**
**THH12_c02 (6,7)**	**25**	**99%**
THH35 01	21	99%
THH35 02	13	99%
THH35 03	12	99%
THH35 04	11	99%
THH35 05	11	99%
THH35 06	11	99%
**THH35_c01 (2,6)**	**24**	**99%**
**THH35_c02 (3,4,5)**	**34**	**99%**

Table S3. **Details of sequenced iL3s that passed our QC and that were included in our bioinformatic analyses.** Host ID and number of iL3s from each host from Bangladesh, Cambodia, Thailand (this study) and for Fiji, Myanmar, Japan and the *S. stercoralis* reference PV001 (all previously published data).

Country	Host ID	Number of iL3s included in analyses
**Bangladesh**	BDH199	4 iL3s
	BDH733	2 iL3s
	BDH742	1 iL3
	BDD31	22 iL3s
	BDD134	4 iL3s
	BDD149	1 iL3
	**3 people**	**7 iL3s**
	**3 dogs**	**27 iL3s**
**Cambodia**	KHH01	1 combined sample
	KHH59	1 iL3 and 1 combined
	KHH83	1 iL3 and 2 combined
	KHD01	1 combined sample
	KHD19	2 combined samples
	KHD32	1 combined sample
	KHD63	1 combined sample
	**3 people**	**2 iL3s, 4 combined**
	**4 dogs**	**5 combined**
**Thailand**	THH01	1 iL3sand 1 combined
	THH04	6 iL3s
	THH10	2 iL3s
	THH12	2 combined samples
	THH21	5 iL3s
	THH35	1 iL3sand 2 combined
	THH56	19 iL3s
	THH61	2 iL3s

	THH63	5 iL3s
	THH75	6 iL3s
	THH76	9 iL3s
	THH82	5 iL3s
	THH130	3 iL3s
	THD141	7 iL3s
	**13 people**	**64 iL3s, 5 combined**
	**1 dog**	**7 iL3s**
**Fiji**	F1	12 iL3s
	F2	1 combined sample
	F3	4 iL3s
	**3 people**	**16 iL3s, 1 combined**
**Myanmar (NCBI PRJDB5112)**	MyHTB10	3 iL3s
	MyHTB122	2 iL3s
	MyHTB177	3 iL3s
	**3 people**	**8 iL3s**
**Japan (NCBI PRJDB5112)**	RK4	2 iL3s
	RK5	2 iL3s
	RK6	2 iL3s
	RK7	2 iL3s
	RK8	2 iL3s
	RK9	2 iL3s
	**6 people**	**12 iL3s**
**Reference (NCBI ERX044031)**	PV001	1 samples (pool of iL3s)
**Total**	**31 people**	**109 iL3s, 10 combined**
	**9 dogs**	**34 iL3s, 5 combined, 1 pool of iL3s (PV001)**
	**Total (excluding the reference)**	**143 iL3s and 15 combined**

Table S4. F_{ST} values among iL3s from different countries and host species. Samples are shown by their country code: BD, Bangladesh; KH, Cambodia, F, Fiji; JP, Japan; MM, Myanmar, TH, Thailand, and by the host code: H, human and D, dog. Dog to dog comparisons are highlighted in orange; dog to human comparisons are highlighted in blue; human to human comparisons are highlighted in green. * shows sympatric human to dog comparisons.

	**BDH**	**BDD**	**KHH**	**KHD**	**FH**	**JPH**	**MMH**	**THH**
**BDH**								
**BDD**	0.39*							
**KHH**	0.19	0.38						
**KHD**	0.19	0.37	0.17*					
**FH**	0.17	0.43	0.15	0.37				
**JPH**	0.31	0.42	0.36	0.31	0.20			
**MMH**	0.16	0.40	0.18	0.22	0.12	0.28		
**THH**	0.14	0.53	0.08	0.62	0.10	0.15	0.07	
**THD**	0.57	0.52	0.56	0.36	0.63	0.63	0.58	0.76*

Table S5. F_{ST} values among iL3s from different countries and host species with sample reduction. Samples are shown by their country code: BD, Bangladesh; F, Fiji; JP, Japan; MM, Myanmar, TH, Thailand, and by the host code: H, human and D, dog. Dog to dog comparisons are highlighted in orange; dog to human comparisons are highlighted in blue; human to human comparisons are highlighted in green. * shows sympatric human to dog comparisons.

	BDH					
BDH		BDD				
BDD	0.37*		FH			
FH	0.17	0.43		JPH		
JPH	0.44	0.42	0.21		MMH	
MMH	0.23	0.40	0.13	0.28		THH
THH	0.08	0.52	0.11	0.17	0.08	
THD	0.55	0.52	0.63	0.63	0.58	0.76*

Table S6. F_{ST}, Admixture and dXY following modified sample inclusion. The sampled iL3s included in analyses were modified as, Modification 1 down-sampled to only a single iL3 per host, though for a single dog host (BDD31) we retained 2 iL3s that were different genotypes; Modification 2 used KING to identify identical iL3s from single hosts, which detected 35 that we then removed. Of these 35, 10 were Japanese samples and 5 Myanmar samples both of which were already published data (23, main text); Modification 3 was as Modification 2, but using dXY rather than F_{ST}; Modification 4 was as Modification 1, but using dXY rather than F_{ST}.

	**F_{ST} or dXY (median values)**	**Admixture**	**Total number of iL3s / Mean / Maximum number of iL3s per host**
**Modification 1**	Dog : Human = 0.65<br>Human : Human = 0.06<br>Dog : Dog = 0.17	k = 8<br>Human only = 4<br>Dog only = 4<br>Shared = 0	34 / 1.1 / 2
**Modification 2**	Dog : Human = 0.43<br>Human : Human = 0.14<br>Dog : Dog = 0.37	k = 7<br>Human only = 2<br>Dog only = 4<br>Shared = 1	124 / 3.2 / 22
**Modification 3**	Dog : Human = 0.255<br>Human : Human = 0.008<br>Dog : Dog = 0.338	n/a	124 / 3.2 / 22
**Modification 4**	Dog : Human = 0.266<br>Human : Human = 0.009<br>Dog : Dog =0.338	n/a	34 / 1.1 / 2

Table S7. Bootstrap support values. For (A) the nuclear clusters in main text **Figure 2B** and where values are percent of 1,000 replicates for the clades indicated, and (B) the mitochondrial clusters in main text **Figure 3A**, and where values are the percent of 500 bootstraps for the clades indicated.

A

Nuclear clades	Bootstrap support
A	100
B	94
C	85
D1	79
D2	82
A (B ,C, D1, D2)	100
B (C, D1, D2)	40
C (D1, D2)	98
D1 (D2)	96

B

Mitochondrial clades	Bootstrap support
1	100
2	100
3	100
4	100
5	100
6	71
7	65
8	100
1 (2,3,4,5,6,7,8)	100
2 (3,4,5,6,7,8)	54
3 (4,5,6,7,8)	51
4 (5,6,7,8)	8
5 (6,7,8)	11
6 (7,8)	24
